# Are children’s stools in Ghana disposed of safely? Evidence from the 2014 Ghana demographic and health survey

**DOI:** 10.1186/s12889-021-10155-7

**Published:** 2021-01-09

**Authors:** Abdul-Aziz Seidu

**Affiliations:** 1 Department of Population and Health, College of Humanities and Legal Studies, University of Cape Coast, Cape Coast, Ghana; 2 College of Public Health, Medical and Veterinary Sciences, James Cook University, Townsville, Queensland Australia

**Keywords:** Children, DHS, Ghana, Public Health, Stool disposal

## Abstract

**Background:**

Safe disposal of children’s faeces has always been one of the main challenges to good hygiene in Ghana. Although it has been proven that children’s faeces are more likely to spread diseases than adults’ faeces, people usually mistake them for harmlessness. This study, therefore, sought to determine the prevalence and factors associated with safe disposal of children’s faeces in Ghana.

**Methods:**

Data from the 2014 Ghana Demographic and Health Survey was used for the analysis. A sample size of 2228 mother-child pairs were used for the study. The outcome variable was disposal of children stools. Both bivariate and multivariable logistic regression analyses were performed to identify the factors with safe child stool disposal.

**Results:**

The prevalence of safe child stool disposal in Ghana was 24.5%. Women in the middle [Adjusted odds ratio (AOR) = 4.62; Confidence Interval (CI) = 3.00–7.10], Coastal Zone [AOR = 4.52; CI = 2.82–7.22], mothers whose children were aged 12–17 [AOR = 1.56; CI = 1.15–2.13] and 18–23 months [AOR = 1.75; CI = 1.29–2.39], and mothers whose household had improved type of toilet facility [AOR = 2.04; CI = 1.53–2.73] had higher odds of practicing safe children’s faeces disposal. However, women from households with access to improved source of drinking water [AOR = 0.62; CI = 0.45–2.7] had lower odds of practicing safe children’s faeces disposal.

**Conclusion:**

Approximately only about 25 out of 100 women practice safe disposal of their children’s faeces in Ghana. The age of the child, ecological zone, the type of toilet facilities, and the type of drinking water source are associated with the disposal of child faeces. These findings have proven that only improved sanitation (i.e. drinking water and toilet facilities) are not enough for women to safely dispose of their children’s faeces. Therefore, in addition to provision of toilet facilities especially in the northern zone of Ghana, there is also the need to motivate and educate mothers on safe disposal of children’s stools especially those with children below 12 months. More so, mothers without access to improved toilet facility should also be educated on the appropriate ways to bury their children’s stools safely.

**Supplementary Information:**

The online version contains supplementary material available at 10.1186/s12889-021-10155-7.

## Background

Access to improved sanitation remains one of the key public health issues in the global agenda, as stipulated in the Sustainable Development Goals (SDGs). For example, Goal 6.2 aims that there is universal access to appropriate sanitation and to end open defecation by 2025 [1]. The World Health Organization (WHO) points out that approximately 1 billion and 2.4 billion people respectively defecate in the open and live without improved sanitation facilities [2]. From these figures, approximately 842,000 people die from health-related diseases each year. Especially in Africa and Ghana, deaths of children under five account for 16 and 25% of diarrhoeal diseases, respectively [3]. In sub-Saharan Africa (SSA), approximately 229 million people engage in open defecation [4]. Approximately, only 21% of the population have access to basic sanitation facilities in Ghana (17% for the rural population and 25% for the urban population) [5]. It is estimated that due to poor sanitation, Ghana loses about 1.6% each year, equivalent to US$290 million of our gross domestic product [5].

In many low- and middle-income countries [LMICs], how to deal with child faeces has always been a problem. It has also received less attention on the agenda of many governments [6–9]. In addition, there is a misunderstanding that children’s faeces are harmless, so most parents dispose of their children’s faeces unsafely [9, 10]. Nevertheless, there is evidence that children’s stools may be at higher risk than adults’ stools, partly because of the presence of diarrhoea and pathogens such as hepatitis A, rotavirus, and *E. coli*. Similarly, children are also severely affected by enteric pathogens, making their stool the main source of infection [8, 11]. Like many LMICs, in Ghana, poor sanitation is one of the main causes of faecal-oral diseases [12, 13].

Evidence again shows that children with unsafe stools are more likely to develop diarrhoea [8, 10]. Based on these substantiations, safe disposal of faeces from children and adults is essential to prevent infection [7, 8, 10, 11, 14–17]. The prevalence of safe disposal of child faeces is 17% in Zimbabwe [18], 19.7% in Nigeria [19], 21.0–27.5% in India [8, 18, 20], and [33.7–36.9% in Ethiopia [7, 11]. The prevalence is also 38.0% in Madagascar, 67.0% in Zambia, 70% in Kenya, 75% in Uganda, and 79% in Malawi [21]. The safe disposal of child faeces is associated with many factors. These include wealth [7, 11], child’ sex, child’s age [7, 11], family size [7], parity, sex of the head of the household [8], mother’s age [11], educational level [7, 8, 19], ecological zone, place of residence [8, 11, 22], ethnicity [8], religion, work status or employment, mother’s exposure to the mass media, the child’s diarrhoea experience [8], ANC attendance, type of toilet facility [7, 8], and source of drinking water [7, 8, 19].

A report by the 2014 Demographic and Health Survey (GDHS) showed that nearly half (47%) of mothers dispose of their children’s faeces by throwing them into the garbage [23]. A small part of the population of Ghana has access to improved toilet facilities [23]. Based on these statistics, greater efforts are needed to strengthen existing strategies aimed at improving health conditions in Ghana. For example, the Ghanaian government has formulated various regional and national policies to improve sanitation conditions. For instance, the “Sama Sama” project in the northern zone (the then Northern and Upper East Regions) signed the “Sanitation and Water for All” project. The government has also drafted various policy documents, including “Environmental Sanitation Policy” [24], “National Environmental Sanitation Strategy and Action Plan” [25] and “Strategic Environmental Sanitation Investment Plan” [26, 27] to help improve sanitation in Ghana.

Although Ghana has adopted many strategies and efforts to improve sanitation conditions, how these strategies affect all parts of the population seems to be unexplored. A typical example is how Ghanaian women dispose of the faeces of  their children. This study is aware of the research gaps and uses nationally representative data to assess the prevalence and associated factors of the safe disposal of children’s faeces by Ghanaian mothers. The results of this study can help formulate measures and strengthen existing policies to promote the safe disposal of children’s faeces in Ghana. 

## Methods

### Description of the survey and sampling

The 2014 edition of the GDHS data was used for this study. GDHS is a nationwide study covering 10 regions at the time, conducted every 5 years. The survey was executed by the Ghana Statistics Service (GSS) and Ghana Health Service (GHS). Inner City Fund International provided technical help through MEASURE DHS. The survey adopts a multi-sampling approach to select the respondents. Particularly, two stages are followed to sample the unit of analysis in the households. In the initial stage, clusters encompassing enumeration areas (EAs) delineated for the 2010 Population and Housing Census were selected from the then 10 administrative regions of Ghana, comprising both urban and rural areas ([23] p.317). Afterwards, households from each cluster are selected and women who fall within these households are interviewed. In the 2014 survey, a total of 9396 women were interviewed (97.3% response rate). The survey provided the complete birth history of women and their children. The current study includes 2228 children under the age of 2 living with mothers in each household. Their mothers answered questions relating to how they dispose of their stools. The GDHS final report [23] provides detailed information on the method, pre-testing, on-site staff training, sampling design, and selection. The report is also available online for free from https://dhsprogram.com/publications/publication-FR307-DHS-Final-Reports.cfm. The study relied on the “Strengthening of Epidemiological Observational Research Report” (STROBE) statement in writing the manuscript (Table S1).

### Ascertainment of variables

#### Outcome variable

Disposal of children’s stool was the outcome variable. It was a binary variable categorised as “safe or unsafe” [7–9, 11, 18–20, 28, 29]. It was derived from the question “The last time [Name] passed stools, what was done to dispose of the stools?” The response included the following: “child used the toilet or latrine,” “put/rinsed into toilet or latrine,” “put/rinsed into drain/ditch,” “thrown into the garbage,” “buried,” “left in the open,” and “other.” The study followed the WHO’s [2] definition of safe disposal of faeces as situations where child used toilet/latrine, ﻿put/rinsed faeces into the toilet or latrine and buried the faeces and unsafe stool disposal as situations where one ﻿put/rinsed faeces into drain or ditch, faeces thrown into the garbage, faeces left or unburied in the open. These responses were recoded as follows: all those who answered “child used toilet or latrine”, buried and those who “put/rinsed into toilet or latrine” were merged and coded as “safe disposal of child stool” (coded as ‘1’) whereas the remaining disposal practices were coded as “unsafe disposal of child stool” (coded as ‘0’).

#### Independent variables

The independent variables were included based on their association with children’s stool disposal in previous studies [7–9, 11, 18–20, 28, 29]. These variables were household wealth (poorest, poorer, middle, richer, richest), sex of child (male, female), age of the child (0–5 months, 6–11 months, 12–17 months, 18–23 months), number of people in household (> 5, < 5), parity (1, 2, 3, 4+), sex of household head (male, female), mother’s age (15–24, 25–34, > 34), mother’s educational level (no education, primary, secondary/ higher), ecological zone (northern, middle, coastal), place of residence (urban, rural), ethnicity (Akan, Ga-Dangbe, Ewe, Mole Dagbani, Others), religion (Christianity, Islam, others), working status (not working, working), mother’s exposure to mass media (newspaper, television, and radio [all captured as, not at all, less than once a week, at least once a week]), child experienced diarrhoea in the last 2 weeks (yes, no), and ANC attendance (none, 1–3 times, 4 or more times). In addition, the toilet facility used by members in the household and source of drinking water for members in the household were all categorized as ‘improved’ and ‘unimproved’ [2, 21].

### Statistical analyses

The analysis of the data followed three key steps. First, the data were extracted from the children’s file and cleaned. Afterwards, frequency counts and percentages were estimated for categorical variables. A chi-square analysis was done to show the factors associated with child stool disposal and those that showed statistical significance (*p *< 0.05) were included in the regression analysis (see Table 1). At the regression analysis stage, both bivariate and multivariable logistic regression modelling were  done and the results were presented as crude odds ratios (COR) and adjusted odds ratios (AOR) respectively with their corresponding 95% confidence intervals (CI) signifying level of precision. Before carrying out the logistic regression analysis, multicollinearity was checked using the Variance Inflation Factor (VIF) (Mean VIF = 1.43, Maximum VIF = 2.35 and Minimum VIF = 1.02). The choice of reference categories was also informed by previous studies and a priori. Sampling weight was assigned at various levels of analysis to account for over- and under-sampling of some areas within the study settings while the svy command was used to take account of the complex sampling procedure.

**Table 1:** Selected socio-demographic characteristics and prevalence of Child’s stool disposal across independent variables among women in Ghana (*N* = 2228)

Variables	Weighted sample		Safe	***P***-values
	N	%		
**Age**				*p* = 0.0856
15–24	568	25.5	22.1	
25–34	1110	49.8	23.2	
35–49	550	24.7	22.5	
**Marital status**				*p* = 0.009
Not Married	807	36.2	26.1	
Married	1421	63.8	21.1	
**Religion**				*P* < 0.001
Christianity	1659	74.4	26.0	
Islam	398	17.9	14.6	
Other	171	7.7	15.8	
**Ethnicity**				*p* < 0.001
Akan	1021	45.8	33.1	
Ga-Dangbme	131	5.9	26.5	
Ewe	295	13.2	30.4	
Mole-Dagbani	429	19.3	13.5	
Others	352	15.8	11.6	
**Wealth**				*p* < 0.001
Poorest	506	22.7	11.6	
Poorer	466	20.9	29.3	
Middle	434	19.5	28.2	
Richer	432	19.4	28.8	
Richest	391	17.5	25.8	
**Residence**				*p* = 0.756
Rural	1229	55.2	22.5	
Urban	999	44.9	23.1	
**Ecological zone**				*p* < 0.001
Northern	451	20.2	6.5	
Coastal	801	36.0	31.9	
Middle	976	43.8	31.0	
**Educational level**				*p* < 0.001
No education	605	27.2	14.1	
Primary	420	18.8	22.4	
Secondary/higher	1203	54.0	29.4	
**Working status**				*p* = 0.032
Not working	477	21.4	26.4	
Working	1751	78.6	21.8	
**Frequency of reading newspaper**				*P* = 0.357
Not at all	1965	88.3	22.3	
Less than once a week	142	6.4	27.6	
At least once a week	119	5.4	24.7	
**Frequency of listening to radio**				*p* = 0.004
Not at all	673	30.2	19.2	
Less than once a week	553	24.8	26.2	
At least once a week	1002	45.0	24.4	
**Frequency of watching television**				*p* < 0.001
Not at all	403	18.1	16.0	
Less than once a week	743	33.3	26.7	
At least once a week	1082	48.6	23.0	
**ANC attendance**				*p* = 0.097
0	65	3.0	13.3	
1–3	242	11.2	20.8	
4+	1860	85.9	23.3	
**Household head sex**				*p* = 0.011
Male	1717	77.1	21.6	
Female	511	22.9	27.1	
**Parity**				*P* = 0.336
1	474	21.3	20.9	
2	487	21.9	25.8	
3	403	18.1	21.9	
4+	864	38.8	22.6	
**Child had diarrhoea**				*p* = 0.302
No	1929	86.6	23.1	
Yes	299	13.4	20.5	
**Sex of child**				*p* = 0.141
Male	1172	52.6	21.5	
Female	1056	47.4	24.1	
**Child’s age (months)**				*p* < 0.001
0–5	545	24.5	17.5	
6–11	586	26.3	20.5	
12–17	565	25.3	25.1	
18–23	533	23.9	28.7	
**Number of household members**				*P* = 0.106
< 5	1290	57.9	24.1	
> 5	938	42.1	21.2	
**Source of drinking water**				*p* < 0.001
Unimproved	293	13.2	11.9	
Improved	1935	86.8	30.7	
**Access to toilet facility**				*p* = 0.028
Unimproved	771	34.6	27.3	
Improved	1457	65.4	21.9	

Source: 2014 GDHS

## Results

### Weighted prevalence of youngest children’s stool disposal in Ghana

**Fig. 1:**
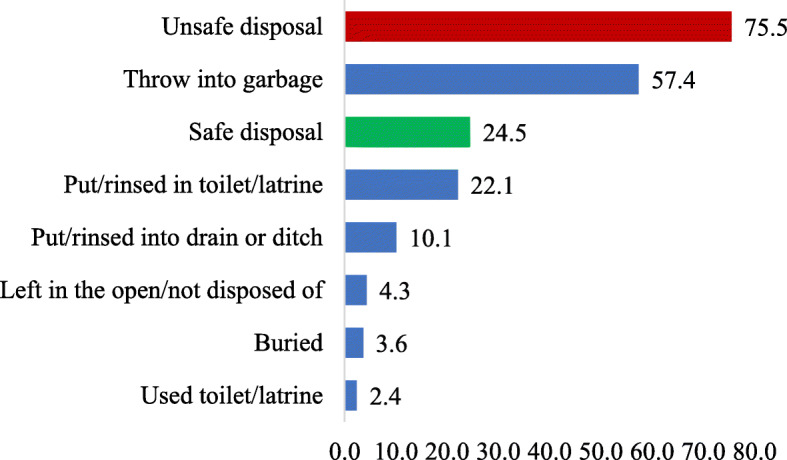
Weighted prevalence of youngest children’s stool disposal in Ghana (*N* = 2228)

Figure 1 presents the weighted prevalence of stool disposal practices among women in Ghana. It was found that a quarter (24.5%) had disposed of their children’s stool safely while 2.4% indicated they disposed it of by using toilet or latrine.

### Prevalence of Child’s stool disposal across independent variables among women in Ghana

Table 1 also shows the background characteristics of the women. It was found that 49.8% were aged 25–34. The majority (63.8%) were married, 74.4% were Christians, 45.8% were Akans, approximately 23% were in the poorest wealth category, and 78.6% were working. Slightly more than half (54.0%) had attained secondary/higher level of education. The majority (55.2%) were also in rural areas while 38.8% had 4 or more children (Table 1).

### Logistic regression results on factors associated with safe children’s stool disposal among women in Ghana

It was found that women in the middle [AOR = 4.62; CI = 3.00–7.10] and coastal zone [AOR = 4.52; CI = 2.82–7.22] had higher odds of practising safe disposal of their children stool, compared to those in the northern zone. The odds of practicing safe child faeces disposal was higher for mothers whose children were aged 12–17 [AOR = 1.56; CI = 1.15–2.13] and 18–23 months [AOR = 1.75; CI = 1.29–2.39], compared to those whose children are less than 6 months. It was also found that mothers whose household had improved type of toilet facility [AOR = 2.04; CI = 1.53–2.73] had higher odds of practicing safe child faeces disposal, compared to those with unimproved toilets. Finally, women from households with access to improved source of drinking water had lower odds [AOR=0.62;CI=0.45–0.85] of practicing safe child faeces disposal, compared to those who had unimproved source of drinking water (Table 2).

**Table 2:** Factors associated with safe child stool disposal among women in Ghana

Variables	COR	95% CI		AOR	95% CI	
		Lower	Upper		Lower	Upper
**Marital status**						
Not Married	Ref	Ref	Ref	Ref	Ref	Ref
Married	**0.76	0.62	0.93	1.12	0.88	1.43
**Religion**						
Christianity	Ref			Ref		
Islam	***0.49	0.37	0.64	0.92	0.64	1.32
other	***0.53	0.36	0.79	1.15	0.74	1.79
**Ethnicity**						
Akan	Ref	Ref	Ref	Ref	Ref	Ref
Ga-Dangbme	0.73	0.45	1.17	0.81	0.50	1.3
Ewe	0.88	0.65	1.20	0.89	0.64	1.24
Mole-Dagbani	***0.31	0.24	0.41	1.08	0.72	1.62
Others	***0.26	0.19	0.37	0.68	0.44	1.03
**Wealth status**						
Poorest	Ref	Ref	Ref	Ref	Ref	Ref
Poorer	***3.17	2.35	4.28	1.17	0.80	1.7
Middle	***3.01	2.19	4.12	1.02	0.66	1.58
Richer	***3.10	2.24	4.29	1.00	0.62	1.61
Richest	***2.66	1.87	3.79	0.72	0.43	1.22
**Educational level**						
No education	Ref	Ref	Ref	Ref	Ref	Ref
Primary	***1.76	1.30	2.38	1.01	0.71	1.44
Secondary/higher	***2.54	1.99	3.24	1.24	0.88	1.76
**Working status**						
Not working	Ref	Ref	Ref	Ref	Ref	Ref
Working	*0.78	0.61	0.98	0.80	0.62	1.03
**Ecological Zone**						
Northern	Ref	Ref	Ref	Ref	Ref	Ref
Coastal	***6.77	4.85	9.45	***4.52	2.82	7.22
Middle	***6.49	4.71	8.95	***4.62	3.00	7.10
**Frequency of watching television**						
Not at all	Ref	Ref	Ref	Ref	Ref	Ref
Less than once a week	**1.495	1.15	1.95	0.79	0.57	1.10
At least once a week	**1.36	1.08	1.71	0.87	0.63	1.20
**Frequency of listening radio**						
Not at all	Ref	Ref	Ref	Ref	Ref	Ref
Less than once a week	**1.91	1.41	2.59	1.32	0.94	1.87
At least once a week	**1.57	1.17	2.10	1.12	0.80	1.56
**Sex of Household head**						
Male	Ref	Ref	Ref	Ref	Ref	Ref
Female	*1.35	1.07	1.71	1.02	0.78	1.33
**Child’s age**						
0–5	Ref	Ref	Ref	Ref	Ref	Ref
6–11	1.21	0.90	1.63	1.06	0.77	1.46
12–17	**1.58	1.18	2.10	**1.56	1.15	2.13
18–23	***1.90	1.43	2.53	***1.75	1.29	2.39
**Type of toilet facility**						
Unimproved	Ref	Ref	Ref	Ref	Ref	Ref
Improved	***3.28	2.61	4.14	**2.04	1.53	2.73
**Source of drinking water**						
Unimproved	Ref	Ref	Ref	Ref	Ref	Ref
Improved	*0.75	0.58	0.97	**0.62	0.45	0.85
Pseudo R^2^						0.12
**N**						**2228**

*p < 0.05, **p < 0.01, ***p < 0.001

*COR* Crude Odds Ratio, *AOR* Adjusted Odds Ratio, *CI* Confidence Interval, *Ref* reference category

Source: 2014 GDHS

## Discussion

### Summary of key findings

This study aimed to assess the prevalence and factors associated with safe disposal of children’s faeces in Ghana. It is probably the first in Ghana that seeks to provide evidence on this phenomenon, using nationally representative data. A quarter (24.5%) of women in Ghana dispose of their children’s faeces safely. The study also found that the type of toilet facilities, the source of drinking water, the age of the child, and ecological zone are the main factors associated with the disposal of children’s faeces. The results of this study are important in Ghana and other low- and middle-income countries with poor sanitation.

### Syntheses with previous evidence

In this study, the prevalence of safe disposal of children’s faeces (24.5%) is similar to results obtained in Bangladesh [28] and India [8, 19, 20] but slightly higher than Zimbabwe [18] and Nigeria [9]. However, it is lower than what is reported in Ethiopia [7, 11], Madagascar [21], Zambia, Kenya, Uganda, Malawi [21], and Nepal [21]. Unsafe disposal of child faeces is more likely to expose children and people around them to various health problems through various mechanisms [11]. Some studies have reported that there is a significant association between unsafe child stool disposal and diarrhoea, soil-borne worm infections, trachoma, and other intestinal diseases [2, 14]. For example, Bawankule et al. [8] revealed that in India, children whose stools were disposed unsafely stood greater chances of suffering from diarrhoea.

Compared with mothers with children 0–5 months old, mothers with children 12 months and older are more likely to safely dispose of their children’s faeces. This result confirms earlier studies in Bangladesh [20, 30] and Ethiopia [7, 11]. Various explanations have been proposed for this association. For example, it has been explained that mothers might think that the faeces of young children are harmless, small, smell less, have fewer visible food residues, and are therefore less disgusting than faeces of adults [10, 30–32]. Another explanation is that as children grow up, they will receive instructions on how to use the toilet by themselves, instead of having their mothers dispose of the faeces [11, 21]. It is, therefore, imperative for community health nurses to educate mothers on the importance of disposing of children’s stools safely irrespective of the child’s age by paying particular attention to those whose children are less than 12 months.

Ecological zone was significantly associated with the disposal of child faeces. It was found that compared with the northern zone (Upper East, Upper West, and Northern Regions at the time), child faeces in the coastal and central zones were more likely to be safely disposed of. This means that mothers in the northern zone are more likely to unsafely dispose of their children’s faeces. Compared with the central and coastal zones, the northern region is dominated by a large number of rural communities. The results can be discussed in the context of the available toilet facilities and the socio-cultural practices surrounding toilet use and the disposal of children’s stools [33], as well as the differences in the disposal of rural and urban children stools as reported in the GDHS report [23], Ethiopia [7, 11], and Kenya [34].

According to data from the World Health Organization [2], in Africa, Ghana is the second-largest open defecation country after Sudan. About 5 million Ghanaians do not have toilet facilities. In 2015, the World Bank also estimated that 18.75% of Ghanaians practice open defecation, which is the most common practice in the northern zone [35]. For example, 89, 72, and 71% of the population in the Upper East Region, North Region, and Upper West Region have no toilet facilities, respectively [2, 33], and most resort to using bushes, fields, or small containers for defecation [33, 36]. In most of these circumstances, children are those who practice open defecation the most since some toilet facilities are originally not designed to easily support their usage [33]. In Ghana, there is an urban-rural gap in sanitation coverage, toilet facilities, and access to safe drinking water. In most cases, some families across the country conform to social norms, beliefs, and expectations regarding hygiene behavioor [22, 33]. For example, Osumanu, Kosoe, and Ategeeng ([33], p.7) found in the northern region (Wa) that people firmly believe that “witches, wizards, and other bad spirits visit the toilet at night and as such woe unto anybody who is spotted by these spirits around those hours in the toilet”. This belief may also prevent mothers from practicing safe disposal of their children’s faeces. Therefore, it is necessary to conduct community publicity through various platforms such as community information centers and social gatherings to educate people about the importance of safe disposal of child faeces.

Another important finding of this study is that mothers with improved toilet facilities in their households are more likely to safely dispose of their children’s faeces. Previous studies in Ethiopia [7], India [8], South Africa [37], and Bangladesh [28] also reported similar findings. As described by Majorin et al. [20], families with toilets are more likely to adopt safer methods of disposing children’s faeces. This is also supported by Sara and Graham [37], who explained that ownership of physical infrastructure of improved sanitation can motivate people to adopt safe hygienic practices. There is, therefore, the need to institute various measures to ensure improvement in the type of toilet facilities in various households.

Similar to previous studies in Ethiopia [7, 11], this study also did not find a statistically significant association with diarrhoea and unsafe disposal of children’s stool disposal. However, there is no information on the frequency of diarrhoea and how easily the questions asked have any connection to unsafe handling. However, unlike previous studies [7, 8, 11, 20], this study found a statistically significant association between the type of drinking water source and the disposal of children’s faeces. Specifically, this study found the opposite relationship, that is, mothers in families with improved water sources are less likely to practice safe disposal of child faeces. This finding contradicts the previous study by Curtis et al. [22]. Possible differences in research results may be variables used to measure the type of water source, differences in settings, and differences in research time. Nevertheless, different types of research designs and methods (such as case-controlled studies or qualitative studies) must be used to clarify these nuances.

### Strength and limitations

The main strength of this study is the use of nationally representative survey data with a relatively large sample size. In this way, it is possible to generalise the findings to women with children under 2 years in Ghana and other geographical locations with similar characteristics. Nevertheless, this study is a cross-sectional study, so it cannot be claimed that the findings obtained have any causal relationship. The outcome variable, the disposal of child faeces, was collected based on reported practice rather than direct observation. Nevertheless, Clarkson et al. [38] found that direct observation is full of Hawthorne effect, so people tend to change their behaviour because they know they are being investigated. There is also the possibility of social desirability and recall bias. However, recall bias is likely to be minimal, given that mothers were asked ﻿the question “the last time [NAME] passed stools, what was done to dispose of the stools?” rather than the usual practice of disposal of stools. Finally, there is the possibility of missing some key variables including hand washing and soap use. 

## Conclusion

This study shows that only a quarter of women safely dispose of their children’s faeces. The age of the child, ecological zone, the type of toilet facilities, and the type of drinking water source are associated with the safe disposal of child faeces. This finding proves that just getting improved sanitation (such as drinking water and toilet facilities) is not enough for women to safely dispose of their children’s faeces. In addition to providing toilet facilities in northern Ghana, health practitioners also need to motivate and educate mothers on how to safely handle children’s faeces, especially mothers with children under 12 months of age. More so, mothers who do not have access to improved toilet facilities must be informed on the safe disposal of their children’s stools. Future studies could look at the disposal practices in the various ecological zones in Ghana, taking into consideration the rural urban divisions as well as the trend of safe disposal practices in Ghana. Qualitave studies are also needed to gain deeper understanding of the reasons why women dispose of their children's stools unsafely. 

## Supplementary Information

**Additional file 1: Table S1.** STROBE 2007 (v4) Statement—Checklist of items that should be included in reports of *cross-sectional studies.*

## Data Availability

Dataset is publicly available via this link https://dhsprogram.com/data/dataset/Ghana_Standard-DHS_2014.cfm?flag=0

## Abbreviations

COR: Crude Adjusted Odds Ratio
AOR: Adjusted Odds Ratio
CI: Confidence Interval
GDHS: Ghana Demographic and Health Survey
SDG: Sustainable Development Goal
WHO: World Health Organisation
SSA: Sub-Saharan Africa
LMICs: Low- and middle-income countries
MLGRD: Ministry of Local Government and Rural Development
GSS: Ghana Statistical Service
GHS: Ghana Health Service
EAs: Enumeration areas
VIF: Variance Inflation Factor
